# Membrane TNF-alpha-activated programmed necrosis is mediated by Ceramide-induced reactive oxygen species

**DOI:** 10.1186/1750-2187-8-12

**Published:** 2013-11-01

**Authors:** Shidrokh Ardestani, Desirae L Deskins, Pampee P Young

**Affiliations:** 1 Department of Pathology, Microbiology and Immunology, Vanderbilt University Medical Center, 1161 21st Avenue South, C2217A MCN, Nashville, TN 37232, USA; 2 Department of Internal Medicine, Vanderbilt University Medical Center, Nashville, TN 37232, USA; 3 Department of Veterans Affairs Medical Center, Nashville, TN 37212, USA

**Keywords:** Membrane-TNF-alpha (mTNF-α), Reactive oxygen species (ROS), Ceramide, Programmed necrosis, Mitochondria

## Abstract

**Background:**

Programmed necrosis is a form of caspase-independent cell death whose molecular regulation is poorly understood. While tumor necrosis factor-alpha (TNF-α) has been identified as an activator of programmed necrosis, the specific context under which this can happen is unclear. Recently we reported that TNF-α can be expressed by human tumor cells as both a membrane tethered (mTNF-α) and a soluble (sTNF-α) form. Whereas low level, tumor-derived sTNF-α acts as a tumor promoter, tumor cell expression of mTNF-α significantly delays tumor growth in mice, in large part by induction of programmed necrosis of tumor associated myeloid cells. In this study we sought to determine the molecular mechanism involved in mTNF-α oxidative stress-induced cell death by evaluating the known pathways involved in TNF receptor-induced programmed necrosis.

**Methods:**

The source of Reactive Oxygen Species (ROS) in mTNF-α treated cells was determined by coculturing RAW 264.7 monocytic and L929 fibroblasts cells with fixed B16F10 control or mTNF-α expressing-melanoma cells in the presence of inhibitors of NADPH and mitochondria ROS. To identify the down-stream effector of TNF-a receptors (TNFR), level of phospho-RIP-1 and ceramide activity were evaluated.

To determine whether mTNF-mediated cell death was dependent on a specific TNFR, cell death was measured in primary CD11b myeloid cells isolated from wild-type or TNFR-1, TNFR-2, TNFR-1 and TNFR-2 double knockout mice, cocultured with various TNF-α isoform.

**Results:**

Tumor derived-mTNF-α increased ROS-mediated cytotoxicity, independent of caspase-3 activity. Although TNFR on target cells were required for this effect, we observed that mTNF-induced cell death could be mediated through both TNFR-1 and the death domain-lacking TNFR-2. ROS generation and cytotoxicity were inhibited by a mitochondrial respiratory chain inhibitor but not by an inhibitor of NADPH oxidase. mTNF-α mediated cytotoxicity was independent of RIP-1, a serine/threonine kinase that serves as a main adaptor protein of sTNF-α induced programmed necrosis. Instead, mTNF-α-induced ROS and cell death was prohibited by the ceramide-activated protein kinase (CAPK) inhibitor.

**Conclusion:**

These findings demonstrate that the mTNF-α isoform is an effective inducer of programmed necrosis through a caspase independent, ceramide-related pathway. Interestingly, unlike sTNFα, mTNF-induced programmed necrosis is not dependent on the presence of TNFR1.

## Background

Tumor necrosis factor-alpha (TNF-α) is an inflammatory cytokine, that activates cell inflammation, proliferation, survival and cell death depending on autocrine/paracrine signals, and on the cellular context [1,2]. The soluble homotrimeric form of TNF-α (sTNF-α) that is released from the cell surface activates multiple signal transduction pathways including NFkB survival pathway. In addition to activation of cell survival pathways, sTNF-α can induce cell death [3,4]. Activation of caspases and initiation of apoptosis has been described as the classic form of TNF-mediated cell death. Recent evidence suggests that sTNF-α can also trigger an alternative form of cell death that is distinct from apoptosis. This form of cell death is referred to as “programmed necrosis” and is dependent on the generation of reactive oxygen species [5,6].

So far, in the majority of studies TNF-mediated programmed necrosis have been attributed to the biological and mechanistic function of sTNF-α and its interaction with TNFR-1 in the presence of pharmacological or genetic inhibition of apoptosis [7,8]. TNF-α can also exist as a membrane-anchored protein [9,10]. Like sTNFα, (mTNF-a) is biologically active and binds either of the two TNF-receptors [11]. A recent study from our laboratory indicated that human lung NSCLC express both soluble and membrane isoforms. Using a murine lung cancer model we showed that unlike sTNF-α, mTNF-α exhibits inhibitory effects on tumor growth and myeloid content. We demonstrated that mTNF-α efficiently induced myeloid cell death through induction of ROS-mediated necrosis in the absence of any apoptosis inhibitors [12]. Currently nothing has been reported on how mTNF-α mediates programmed necrosis.

Soluble TNF-induced programmed necrosis typically occurs where apoptosis is inhibited, and is mediated through a few defined pathways. In all cases, the serine/threonine kinase receptor interacting protein-1 (RIP-1) has been shown to play a central role in initiation of programmed necrosis [13,14], mainly through nicotinamide adenine dinucleotide phosphate (NADPH) oxidase (NOX) or mitochondria [15-18]. Recent studies have also described a role for ceramide-mediated programmed necrosis. An increase in the level of intracellular ceramide has been linked to increased redox reaction within the cell [19,20], suggesting the potential for crosstalk between ceramide, ROS, and TNF-α pathways in this process. In spite of these observations, the specific mechanism by which ceramide signaling leads to increased redox reactions is not fully understood.

In this study we sought to determine the molecular pathway involved in mTNF-α-mediated oxidative stress-induced cell death. Using inhibitors targeting mitochondrial electron transport chain and NADPH oxidase we concluded that mitochondrial-dependent oxidative stress was the major source of mTNF-induced intracellular ROS generation, regulated by ceramide-activated protein kinase (CAPK) activity. To our knowledge, this is the first report identifying mTNF-α isoform as a potent activator of programmed necrosis, even in the absence of inhibitors of apoptosis, via ceramide-dependent mitochondrial ROS generation.

## Results and discussion

### mTNF-α is an inducer of cell death

**Figure 1:**
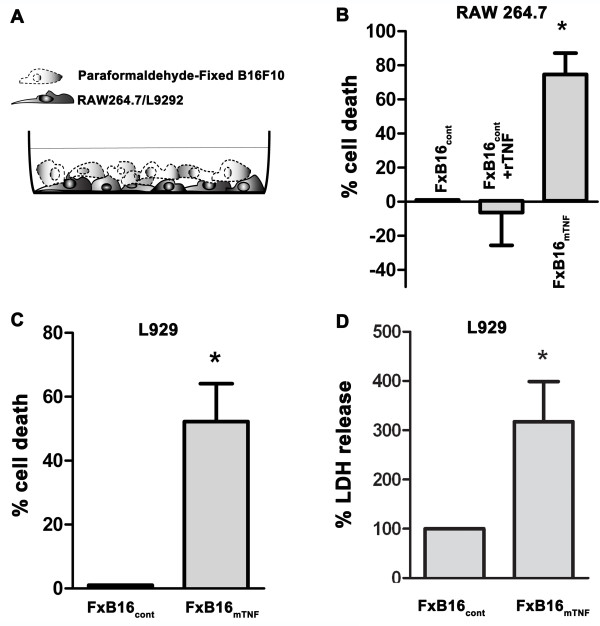
**Membrane TNF-α isoform effectively induces cell death. A***,* Schematic diagram of fixed B16F10 cells cocultured with L929/RAW264.7. **B***,* RAW cells were cocultured with paraformaldehyde-fixed control (FxB16cont), control + rTNFα (FxB16cont + TNFα), or mTNFα (FxB16mTNF) for 24 hours. Cell death was measured by MTT assay. **C***,* MTT assay showing the cytotoxic effects of mTNF-α isoform on L929. **D**, LDH assay measuring L929 cells percent LDH leakage, in the presence of control or mTNF-expressing fixed B16F10 tumor cells. Data show the percentage of LDH leakage into media to total LDH (media + cells). Each sample was assayed in triplicate, with each experiment repeated at least 3 times independently. Data are expressed as average ± S.E. *P < 0.05 and **P < 0.005, 1-way ANOVA of log (10) transformed data with Student’s t-test as post-test (*B*), students’s *t-test* (*C and D*).

To investigate the ability of the membrane versus soluble TNF-α isoforms to induce cell death, RAW 264.7 cells, a line derived from murine leukemic monocytes/macrophage cells (target), were mixed with 1% paraformaldehyde-fixed B16F10 melanoma cells (effector) expressing empty vector, ±100 U/ml rTNF-α (FxB16_cont_ or FxB16_cont_ + rTNF), or fixed mTNF-expressing B16F10 cells (FxB16_mTNF_) at a target: effector ratio of 1:10. Paraformaldehyde-fixed tumor cells were used to eliminate the endogenous sTNF-α (Figure 1A). As measured by the MTT dye reduction assay, incubation with FxB16_mTNF_ resulted in more than 70 ± 12% cytotoxicity of RAW 264.7 myeloid cells after 48 hours of incubation, as compared to RAW 264.7 cells incubated with FxB16_cont_ (Figure 1B, *P* < 0.05). In contrast FxB16_cont_ + rTNF increased RAW 264.7 cell survival compared to control.

To determine the molecular pathway leading to mTNF-induced cell death, we utilized the highly TNF-sensitive L929 fibrosarcoma cell line. As shown in Figure 1C, mTNF-α isoform resulted in more than 50% cell death compared to L929 cocultured with FXB16_cont_ as determined by MTT reduction assay. Cellular toxicity causes membrane damage and results in the release of lactate dehydrogenase (LDH) from the cytoplasm and thus LDH in the media can be used to measure cell death. To confirm the results obtained with MTT assay, we measured LDH release in L929 cell in the presence of control or mTNF-expressing tumor cells. The mTNF-α isoform increased the level of LDH leakage by 3.7 fold over control (Figure 1D).

### Membrane TNF-α-induced cell death can be mediated through both TNFR-1 and TNFR-2

**Figure 2:**
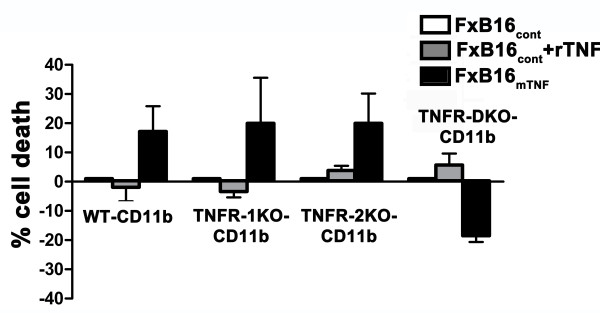
**Membrane TNF-induced cell death can be mediated through both TNFR-1 and TNFR-2.** Freshly isolated CD11b myeloid cells from wild type mice (WT-CD11b), mice deficient in TNF receptor 1 (TNFR-1KO-CD11b), TNFR-2KO-CD11b or both receptors (TNFR-DKO-CD11b). Cells were cocultured with paraformaldehyde-fixed control (FxB16cont), control in the presence of 100 U/ml recombinant TNFα (FxB16cont + rTNF), or mTNFα (FxB16mTNF) for 24 hours. Percentage of cell death was measured by MTT assay. Data present mean percentage (bars, mean ± S.E.) of three replicates from 3 independent experiments.

mTNF-α signal transduction has been linked to a cooperative signaling between TNFR-1 and TNFR-2 [7,21]. Next we sought to determine whether mTNF-mediated cell death was dependent on a specific TNF receptor. Primary CD11b myeloid cells were isolated from wild-type (WT), TNFR-1 knockout (TNFR-1KO), TNFR-2KO or TNFR-1 and TNFR-2 double knockout (TNFR-DKO) and cocultured with fixed control tumor cells with or without rTNF or fixed mTNF-expressing tumor cells for 48 hours. Cell cytotoxicity was determined by MTT assay. As shown in Figure 2, presence of either TNFR-1 or TNFR-2 resulted in increased levels of mTNF-induced cytotoxicity similar to WT-CD11b (~17% cell death in WT-CD11b, ~20% in TNFR-1KO-CD11b and TNFR-2KO-CD11b). In contrast, control cells treated with rTNF improved cell survival in WT- and TNFR-1KO-CD11b and resulted only in ~4% cell death in TNFR-2KO-CD11b and ~5% cell death in TNFR-DKO-CD11b. Interestingly, mTNF-mediated cell cytotoxicity was reversed in TNFR-DKO-CD11b cells. These findings suggested that mTNF-induced cell death can be mediated through both TNFR-1 and TNFR-2.

### Membrane TNF-α exerted cell cytotoxicity by increasing intracellular ROS production

**Figure 3:**
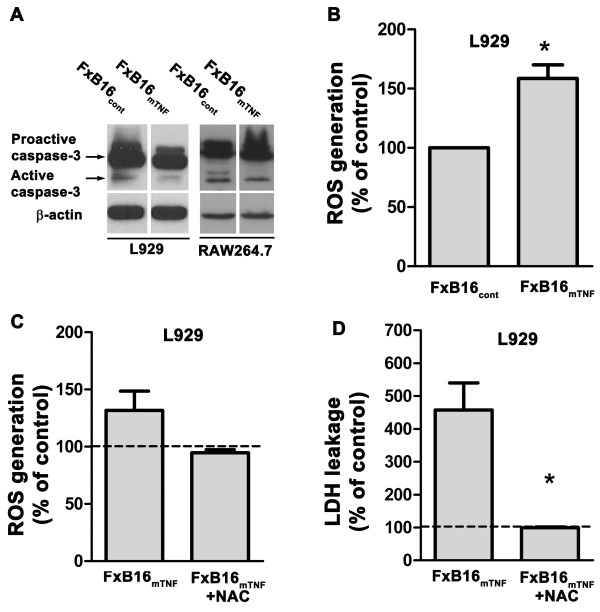
**Membrane TNF-α exerts cell cytotoxicity by increasing intracellular ROS production. A***,* caspase-3 activity in L929 and RAW 264.7 cells after incubation with paraformaldehyde-fixed control (FxB16cont) or mTNFα (FxB16mTNF) for 30 minutes. L929 cells were harvested and total cellular protein was analyzed for active caspase-3. **B***,* ROS production measured by CM-H2CDFDA intensity in L929 cocultured with fixed control or mTNF-expressing B16F10. **C***and***D**, Addition of N-acetyl cysteine (2 mM) reduced ROS level **(C)** and decreased LDH leakage into the media **(D)** in L929 cells. Data are represented as percent of CM-H2DCFDA intensity **(C)** or LDH in media/total LDH **(D)** to L929 cells cocultured with FxB16cont cells with or without NAC. Data are expressed as average ± S.E. *P < 0.05 and **P < 0.005.

Induction of cell death by sTNF-α occurs mainly through activation of caspases leading to apoptosis. To test whether the mTNF-α isoform exerts its cell toxicity in part by activating the caspase pathway, we determined the level of cleavage/activation of caspase-3 proteins in L929 cells treated with fixed control or mTNF-expressing tumor cells. As presented in Figure 3A, treatment of L929 cells or RAW cells with FxB16_mTNF_ did not result in an increased level of active-caspase-3.

Previously we have shown that mTNF-α-treated myeloid cells exhibit increased intracellular ROS and decreased cell survival [12]. To demonstrate an association between intracellular ROS level and cell death in L929 fibrosarcomas, cells incubated with different TNF-α isoforms were measured using CM-H2DCFDA (which quantitatively reacts with oxygen species to produce a highly fluorescent dye). L929 cells incubated with FxB16_mTNF_ resulted in a 60% increase in CM-H2DCFDA fluorescence, indicating an increase in the level of ROS (*P* > 0.05; Figure 3B). Furthermore, incubation of L929 cells with ROS scavenger N-acetyl-cysteine (NAC) reduced mTNF-mediated ROS level (130% of control in FxB16_mTNF_; 94% of control in FxB16_mTNF_ + NAC; Figure 3C, *P* < 0.5). This was followed by 4-fold decrease in LDH release in mTNF-treated L929 cells supplied with NAC (*P* < 0.05; Figure 3D).

### Inhibition of mitochondrial respiratory chain decreased mTNF-mediated ROS generation

**Figure 4:**
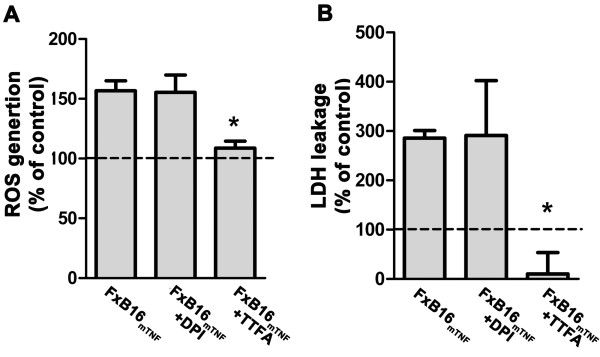
**Inhibition of mitochondrial respiratory chain decreases mTNF-mediated ROS generation. A***and***B***,* L929 cell cocultured with mTNF-expressing tumor cells in the absence or presence of NOX inhibitor-DPI (2 μM) and mitochondrial complex II inhibitor-TTFA (0.5 μM) for 24 hours. TTFA reduced ROS level, shown by reduction of CM-H2DCFDA intensity **(A)** and LDH leakage into media **(B)**. Addition of NOX inhibitor-DPI had no effects on both ROS generation of LDH level. Data are represented as % of CM-H2DCFDA intensity **(A)** or LDH in media/total LDH **(B)** to L929 cells cocultured with control expressing tumor cells with or without DPI or TTFA. Data are expressed as average ± S.E. *P < 0.05.

Next we sought to determine the source of ROS in response to mTNF-α in L929 cells. There is report of nicotinamide adenine dinucleotide phosphate (NADPH) oxidase-1 (NOX1) and mitochondria as the two major source of TNF-α induced ROS production [22,23]. To do so, the NADPH-dependent oxidase and the mitochondrial respiratory chain complex II were blocked using DPI (2 μM) and TTFA (0.5 μM) respectively. The NADPH-dependent oxidase inhibitor DPI did not inhibit the ROS production induced by mTNF-α (156.7 ± 8.3% of control in FxB16_mTNF_ and 155.4 ± 14.4% in FxB16_mTNF_ + DPI; *P* < 0.05; Figure 4A) and further had no effect on the LDH leakage (270% of control in FxB16_mTNF_ and 280% in FxB16_mT_ + DPI; *P* < 0.05; Figure 4B). However, addition of TTFA into L929 cells cocultured with mTNF-expressing tumor cells, reduced CM-H2DCFDA oxidation (108% of control) and LDH release (10% of control, Figure 4B). These data suggested that mitochondria are the source of mTNF-induced ROS generation and cell death.

### Membrane TNF-mediated ROS production involves ceramide pathway

**Figure 5:**
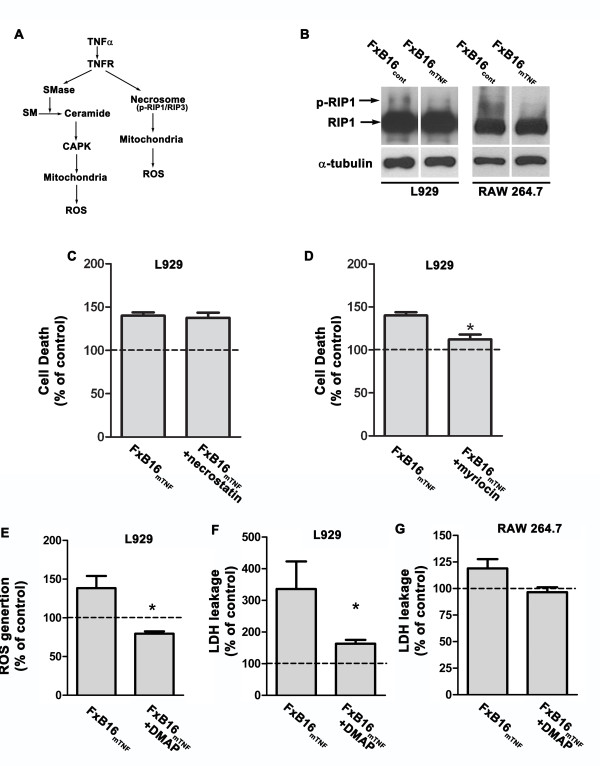
**Membrane TNF-mediated ROS production involves ceramide pathway. A***,* Schematic diagram of TNF-activated pathways leading to ROS generation. **B***,* level of phospho-RIP1 in L929 or RAW 264.7 cells treated with fixed B16F10 control cells (FxB16cont) or B16F10 mTNF (FxB16mTNF) cells. After 30 min incubation, L929 cells were harvested and total cellular protein was analyzed for RIP-1. **C**, inhibition of Rip-1 caused no change in L929 cell death as measured by MTT assay **D**, inhibition of ceramide synthesis with myriocin decreased mTNF-mediated L929 cell death. **E-G***,* inhibition of CAPK reduced mTNF-mediated ROS generation **(E)** and LDH release in L929 **(F)** and LDH release in RAW26.7 **(G)**. Data are expressed as average ± S.E. *P < 0.05.

TNFR-mediated mitochondrial ROS generation can be induced through RIP-1 kinase activity or through a ceramide-dependent signaling pathway initiated by SMase activity (Figure 5A). To determine the specific pathway responsible for activation of mitochondrial ROS production we analyzed level of active RIP1 by evaluating its phosphorylation in L929 or RAW 264.7 in the presence of FxB16_cont_ or FxB16_mTNF_ by immunoblot analysis. As shown in Figure 5B, treatment of both cell lines with fixed membrane expressing tumor cells, FxB16_mTNF_, did not increase the level of RIP-1 phosphorylation. To further confirm that the RIP-1 kinase signaling pathway is not involved in the generation of mitochondrial ROS we assessed cell death by MTT assay with L929 cells incubated with fixed tumor cells, with or without the RIP-1 inhibitor necrostatin (Figure 5C). The inhibitor did not cause any change in L929 cell death when incubated with FxB16_mTNF_ compared to control.

There is evidence to support the role of ceramide as a second messenger of TNF-α activated cells involved in activation of programmed necrosis [24]. Next we evaluated the role of ceramide signaling in TNF-induced ROS production and survival. Addition of myriocin, a ceramide inhibitor, reduced the cell death seen in L929 cells incubated with FxB16_mTNF_ to a level similar to that seen with cells incubated with control tumor cells (Figure 5D). Furthermore addition of DMAP (1 mM), a CAPK inhibitor, reduced mTNF-induced ROS by 60% (138 ± 15.6% of control in FxB16_mTNF_; 80 ± 2.9% in FxB16_mTNF_ + DMAP; Figure 5E). Percentage of LDH leakage was also reduced from 276% in mTNF-treated cells to 163% in mTNF-treated cells supplied with DMAP (*P <* 0.005, Figure 5F). These findings suggested that mTNF-induced mitochondrial ROS generation requires protein kinase activity associated with ceramide. This was further confirmed in RAW 264.7 cell lines. Similar to L929 cells, inhibition of CAPK in mTNF-α treated Raw 264.7 cells reduced LDH leakage (Figure 5G).

## Conclusions

Previously necrotic cell death has been defined as a sudden, unregulated form of cell death which leads to inflammation and tissue damage. However, in recent years accumulating evidence suggests that not all form of necrotic cell death is accidental but can instead be a programmed event [5,6]. There have been several reports of TNF-induced programmed necrosis, mainly in the context of the soluble form of TNF-α. Importantly, induction of programmed necrosis by sTNF-α typically requires the presence of inhibitors of caspases [25,26]. Here, we present for the first time that the lesser-known membrane form of TNF-α has the ability to induce programmed necrosis through ROS generation, independent of caspase inhibitors. In this study we explored the mechanism of mTNF-mediated ROS generation and programmed necrosis.

In our study treatment of mTNF-induced L929 cells with mitochondrial inhibitor complex II increased ROS reduction and improved survival, suggesting a role for mitochondrial complex II in mTNF-mediated programmed necrosis. The plasma membrane-associated NADPH oxidases (NOX) have been proposed as an alternate source of ROS production [15,17,27]. In contrast to what we observed with mitochondrial inhibitor, inhibition of NOX failed to inhibit ROS generation and to increase cell viability.

Although, RIP1/RIP3 kinases have been shown to orchestrate the programmed necrosis pathway activity of sTNF-α, we did not detect any phosphorylated RIP1; instead we found that ceramide pathway was involved in mTNF-α-induced cell death. Ceramide pathway has been identified as an alternative mechanism for induction of programmed necrosis [13,28]. An enhanced level of ceramide has been shown to contribute to depletion of mitochondrial reduced glutathione [29] and increasing mitochondria susceptibility to GD3, a ceramide-derived ganglioside. GD3 traffics to the mitochondria and directly induces ROS production [30,31]. In our study mTNF-induced ROS and cell death seems to be regulated through activity of ceramide since the inhibitor of CAPK, blocked mTNF-mediated ROS and cell death.

The molecular mechanism of the two different TNF-α isoforms remains elusive. It is interesting that although sTNF-α and mTNF-α have similar structures and are able to interact with both TNF-α receptors, they exert opposing effects on tumor growth and cell survival. This raises questions about the plausible molecular mechanisms to account for this difference.

The variation in biological response of soluble versus the membrane form of cytokines has been observed in other members of the TNF receptor superfamily. Membrane form of Fas ligand, for example, has been shown to have opposing role in modulating cell death when compared to its soluble form [32]. Previous work by Gregory and colleague has shown that in glaucoma, full-length FasL accelerates retinal ganglion cells death. By contrast, FasL-deficiency, or administration of soluble FasL, has a protective effect [33]. TNF-related apoptosis-inducing ligand (TRAIL), which has important functions in inducing apoptosis, has shown to have differential activation capacity toward TRAIL-R1 and R2 as a soluble form versus membrane form [34].

Unlike soluble TNF-α which its signal transduction is mediated mainly through TNFR-1 [5,35], it is evident from our study that mTNF-α is efficient in activation of both TNFR-1 and TNFR-2. Members of the tumor necrosis factor receptors superfamily (TNFR-1, Fas, TRAIL) that are capable of inducing cell death usually contain a conserved DD in the intracellular region that is required for activation cell death [1,36]. It is interesting that in our study TNFR-2 which lacks a DD-domain, also seems to be sufficient for induction of mTNF-α-mediated cell death. This phenomenon has been reported in other studies. For instance, TNF-R2 shown to trigger cell death in the rhabdomyosarcoma cell line KYM-1 [37] and the stimulation of CD30 induces cell death in T cell hybridomas [38]. It is not yet clear how TNF receptor superfamily members lacking a death domain (i.e. TNF-R2) execute their death inducing capability. This effect could be FADD-dependent [39] or could be mediated through cooperative activity with other receptors such as Fas/FasL [40].

It has been proposed that membrane anchored TNF-α creates a more stable contact with TNFR1 or TNFR2 with longer half-life. This will result in the formation of higher-order receptor complexes (receptor clustering) [41]. These clusters of receptors are formed in lipid rafts abundant in sphingomyelin which are the precursor of ceramide [42]. Formation of the raft and receptor clustering recruit sphingomylinases to the membrane and result in increased levels of ceramide. Ceramide will decrease mitochondrial membrane integrity and ROS leakage [43]. It is possible that mTNF-α-mediated ceramide production is regulated through multiple TNF receptor superfamily members clustering. This requires further investigation.

In conclusion we have demonstrated that mTNF-α can induce cell death independent of caspase inhibitors by increasing ROS. This occurs through RIP-1-independent, ceramide-dependent activation of mitochondrial ROS. Molecular mechanism, leading to mTNF-induced ceramide formation and mitochondrial-ROS generation, remains to be investigated.

## Methods

### Mice, cell lines and materials

Wild-type C57Bl/6 (WT) mice were purchased from Jackson Laboratory. Homozygous mutants for TNFR-1, TNFR-2, and TNFR-1/2 double knockout (TNFR-DKO) on a C57Bl/6 background were a generous gift from Dr. D. Polk. B16F10 melanoma, RAW264.7 and L929 cells were purchased from American Type Culture Collection (ATCC) and were maintained in DMEM with 10% (v/v) fetal bovine serum, heat inactive fetal bovine serum and MEM with 10% (v/v) horse serum, respectively. N-Acetyl-L-cysteine (NAC), 2-Thenoyltrifluoroacetone (TTFA), myxothiazol and 4-(Dimethylamino)pyridine (DMAP) were purchased from Sigma-Aldrich and prepared fresh on the day of the experiment.

### Constructs

Membrane TNF-expressing cells were generated by cloning the mTNFΔ1-9, K11E sequence encoding a mutant transmembrane TNF-α protein with a deletion at the cleavage site between pre-sequence and mature membrane TNF-α (BCCM/LMBP plasmid collection, Ghent University) into the BamH1-EcoR1 site of LZRS-IRES-Neo retroviral vector, conferring neomycin resistance. This mutation prevents cleavage of the 26-kDa membrane TNF-α into secretory TNF-α isoform. An empty LZRS vector was used as a control vector [12].

### Surface expression of TNF-α

Trypsinized cells were incubated with anti-TNF-α antibody (1 μl/2.5 × 10^4^ cells, Southern Biotech) for 30 minutes on ice. PE conjugated secondary antibody (0.125 μg/10^6^ cells/100 μl) was added for 30 minutes on ice. Surface expression of TNF-α was measured using flow cytometry.

### TNF-α cytotoxicity assay

Overnight cultured RAW 264.7, L929 or freshly isolated bone marrow CD11b cells (EasySep, StemCell Technologies) (2.5 × 10^4^/100 μL/well, target) were cocultured with Paraformaldehyde-fixed [44] control, control + 100 U/ml of recombinant TNF-α or mTNF-α (effector) at target:effector ratio of 1:10 and incubated for 48 hours. Cells were labeled with 100 μl of PBS containing 0.5 mg/mL of 3-[4,5-dimethylthiazol-2-yl] 2,5,-diphenyltetrazolium bromide (MTT) (Sigma) for 2 hours at 37°C then lysed with 0.1 ml DMSO. Photometric measurement was carried out at 540 nm. Percentage of cell death was calculated by using the following formula: Cell death (%) = (1 – OD_sample_/OD_control_) x 100.

### Lactate dehydrogenase (LDH) assay

Early cell damage was determined using the LDH cytotoxicity detection kit (Promega, Madison, WI), which quantifies the LDH release from the cells into the culture medium. Cells were seeded in a 96-well plates at a density of 2.5 × 10^4^ cells/well overnight to promote adherence. Cells were cocultured with fixed B16F10 control or mTNF-α in the absence or presence of the indicated treatments for 24 hours. Supernatants from the cultures were collected and used in the LDH assay as instructed by the manufacturer. LDH activity was detected separately in the supernatant and cell lysate. The percentage of LDH leakage was calculated as 100 x (LDH _supernatant_ / (LDH _supernatant + lysate_)).

### Measurement of intracellular ROS

The oxidant-sensing probe CM-H2DCFDA (Invitrogen) was used to detect intracellular reactive oxygen species (ROS). An overnight culture of cells (2.5 × 10^4^) were loaded with 10 μM CM-H2DCFDA for 30 minutes and cocultured with 2.5 × 10^5^ fixed B16F10 control or mTNF-expressing cell in the absence or presence of the indicated treatments for 6 hours. Fluorescence was determined using a luminescence spectrophotometer (Spectra max, Molecular Devices) with an excitation wavelength of 495 nm and emission wavelength of 525 nm.

### Immunoblot analysis

Target cells were plated at a density of 10^6^ cells overnight (37°C, 5% CO_2_). Cells then were treated with fixed control or mTNF-expressing B16F10 at target cell/fixed tumor cell ratio of 1:10. After 30 minutes incubation at 37°C, target cells were lysed with RIPA buffer [3 M NaCl, 1 M Tris, 0.5 M EDTA, 10% SDS, 1% NP40 substitute and 1× Complete Protease Inhibitor Cocktail (Roche)]. Whole cell lysate was evaluated for caspase-3 cleavage (Cell Signaling Technology) and RIP-1 (BD biosciences) by Western blot analysis.

### Statistical analysis

The statistical significance between experimental and control groups was determined by performing log (10) transformation of the data such that we can apply the parametric analysis, ANOVA followed by Student’s t-test as a post-test using Prism software (Graphpad, San Diego, CA). A *P*-value of <0.05 was considered statistically significant.

## Abbreviations

LDH: Lactate dehydrogenase; TTFA: Thenoyltrifluoroacetone; CAPK: Ceramide-activated protein kinase; DPI: Diphenyleneiodonium; DMAP: 4-Dimethylaminopyridine.

## Competing interests

The authors declare that they have no competing interests.

## Authors’ contributions

SA contributed in design, development of methodology, acquisition of data, and writing of the manuscript. DD contributed in design and acquisition, analysis and interpretation of data. PPY contributed in design, development of methodology, analysis and interpretation of data and writing of the manuscript and supervised the study. All authors read and approved the final manuscript.
